# Risk factors for violent behaviors in patients with schizophrenia: 2-year follow-up study in primary mental health care in China

**DOI:** 10.3389/fpsyt.2022.947987

**Published:** 2023-01-20

**Authors:** Zhuo-Hui Huang, Fei Wang, Zi-Lang Chen, Yao-Nan Xiao, Qian-Wen Wang, Shi-Bin Wang, Xiao-Yan He, Christine Migliorini, Carol Harvey, Cai-Lan Hou

**Affiliations:** ^1^Guangdong Mental Health Center, Guangdong Provincial People's Hospital (Guangdong Academy of Medical Sciences), Southern Medical University, Guangzhou, Guangdong, China; ^2^Luoding Mental Health Center, Yunfu, Guangdong, China; ^3^Peking University Sixth Hospital, Peking University Institute of Mental Health, NHC Key Laboratory of Mental Health (Peking University), National Clinical Research Center for Mental Disorders (Peking University Sixth Hospital), Beijing, China; ^4^Liuzhou Worker's Hospital, Liuzhou, Guangxi, China; ^5^Psychosocial Research Center, University of Melbourne, Melbourne, VIC, Australia; ^6^North Western Mental Health, Melbourne, VIC, Australia; ^7^School of Medicine, South China University of Technology, Guangzhou, Guangdong, China

**Keywords:** violence, schizophrenia, risk factor, primary care, Generalized Estimating Equations

## Abstract

**Objective:**

The consequences and impact of violent behavior in schizophrenia are often serious, and identification of risk factors is of great importance to achieve early identification and effective management.

**Methods:**

This follow-up study sampled adult patients with schizophrenia in primary mental health care in a rural area of southern China, in which 491 participants completed a comprehensive questionnaire at baseline and the 2-year follow-up. Sociodemographic, clinical and psychological assessment data were collected from all participants. Paired sample *T-*Tests and the McNemar Test were performed to examine changes over the follow-up period. Generalized Estimating Equations (GEE) were used to analyze the risk factors for violent behavior.

**Results:**

The results showed that about two in five community-dwelling patients with schizophrenia reported violent behavior in the past year. At follow-up, participants were significantly less employed, had more times of hospitalization, more psychotropic medication, and severer depressive symptoms, but had better health-related quality of life than at baseline. Use of clozapine and better insight into medication decreased the possibility of violent behavior, while more severe positive symptoms, insomnia, as well as use of second-generation antipsychotics other than clozapine, antidepressants and mood stabilizers increased the possibility of violent behavior.

**Conclusions:**

Risk evaluation, prevention and management of violence in patients with schizophrenia are demanded in primary mental health care.

## 1. Introduction

Violent behavior in patients with schizophrenia or other psychosis is a research interest dating back over six decades (1). Violence is a broad concept, including everything from verbal threats and hostility to homicide. Fazel et al. (2) defined violent behavior as homicide, assault, robbery, arson, any sexual offense, or illegal threats and harassment, while Rund's study focused on severe violence (3). The lack of clear operationalized definitions of violent behavior applied in different research has led to mixed results. In general, the causes of violent behavior are multifactorial, including individual as well as social or environmental factors, such as substance abuse, past violent victimization, violence in the surrounding environment and so on (3–5). Violence in psychiatric disorders might be a multidimensional issue which includes several subtypes with different psychopathological underpinnings, and is often linked to acute psychomotor agitation than a long-lasting state (48, 49).

Despite many inconsistent findings, it is generally agreed that there is a modest association between violence and schizophrenia or other psychosis (6, 7). Meta-analysis has revealed the odds of homicide being committed by individuals with psychosis was almost 20 times that of the general population irrespective of comorbid substance abuse (8). A further meta-analysis also demonstrated that schizophrenia spectrum disorders are associated with a heightened risk of violent offending (9), in which some studies estimated that up to 20% of patients with schizophrenia in the community will behave violently in a 6-month period (34).

The consequences of violent perpetration among people with schizophrenia or other psychosis are often very serious for the victims, the patient themselves and the wider community (3), which leads to serious public health concern and social problems (10). Furthermore, the high disease burden and economic impact associated with violence are also critically important. Senior et al. (11) noted that the estimated annual economic impact of violence perpetrated by people with severe mental illness was £2.5 billion in England and Wales in 2015 to 2016, or 5.3% of the total estimated societal cost of violence. Unfortunately, no relevant Chinese data of economic impact of violence perpetrated by patients with schizophrenia or other severe mental illness.

Therefore, exploration of risk factors for violent behavior in schizophrenia or other psychosis is of great importance, so as to achieve early identification and prevention. A previous review found that severe violence appears particularly associated with poor insight, high impulsivity, psychopathy, poor motor speed and global cognition (3). Fazel et al. (2) reported that the strongest predictors for violent offending within 12 months in patients with severe mental illness were conviction for previous violent crime (adjusted OR = 5.03) and male sex (adjusted OR = 2.32), and meanwhile the decline in probability of violent offending was linearly related to increasing age (adjusted OR = 0.63 per 10 years of age). Similarly, an Italian study showed that patients with severe mental illness with a history of violence had a greater frequency of lifetime domestic violence than those without a violence history (12).

However, many previous studies examining risk factors for violence in psychosis tend to be cross-sectional in design and conducted in well-developed regions (4, 12–14). Therefore, this study aimed to explore the socio-demographic features, clinical characteristics, and medication of patients with schizophrenia using a longitudinal design. We hypothesized that severer psychiatric symptoms, severer depressive symptoms, poorer insight into disease and insomnia will increase the possibility of violent behavior.

In particular, the design will enable the identification of risk factors for their violent behavior in a rural area of the middle-income country of China, thus enhancing the generalizability of the results to similar developing regions. A secondary aim of the study is to strengthen risk evaluation and guide prevention, as well as management, of potentially dangerous behaviors in patients with schizophrenia or other psychoses in this and similar settings.

## 2. Methods

### 2.1. Study design and participants

This follow-up study was led by Guangdong Mental Health Center, Guangdong province in China, and The University of Melbourne. Ethical clearances were obtained from the above-mentioned institutions (Z2019-120 and 2021-20740-15415-3, respectively).

Study participants were all administered by, and recruited from Luoding city, which is an underdeveloped and rural area of Guangdong province in southern China, fitting the research interest of the present study. Also, Luoding is a city which can provide relatively good primary mental health services in Guangdong Province. In the present study, 21/63 townships with primary mental health care services in Luoding city were chosen by a random cluster sampling method, for which the randomized digital table was applied. All local patients with schizophrenia who presented to primary mental health care services were registered and managed in the Chinese National Psychiatric Management System (CNPMS). CNPMS was established to provide community follow-up management for severe mental illness, in which individuals with schizophrenia, schizoaffective disorder, paranoid psychosis, bipolar disorder, mental disorder related to epilepsy, and intellectual disability with psychotic symptoms were required to enroll.

First, the sampled schizophrenic patients from CNPMS were contacted by telephone to clarify the details of the research protocol. If they were willing to participate, both participants and their caregivers would took part in a 1-h face-to-face interview, which was conducted by one of three psychiatrists, each with no <3 years clinical and scientific research experience. After the interview, patients were included if they met the inclusion criteria (see below for more details) for the present study. At baseline, a total of 742 patients with schizophrenia were included. The baseline survey was conducted from October 2015 to January 2016 (15). 2 years later, researchers telephoned all the study participants and conducted face-to-face follow-up evaluations from November 2017 to January 2018, in which 491 participants completed the interview, and the remaining 251 participants refused follow-up. We only conducted one follow-up till now, yet there will be on-going follow-ups in the future.

Inclusion criteria for the participants were as follows: (a) aged 18 years or older; (b) diagnosed with schizophrenia (according to ICD-10) based on a review of the medical record and supplemented by clinical interview conducted by one psychiatrist; (c) capable of understanding and completing the interview according to the researchers. Exclusion criteria were having a history of significant head injury, seizures, cerebrovascular diseases and other neurological diseases.

Each eligible potential participant who was deemed competent to participate in an interview was informed of the purpose, significance, content (inclusion criteria, research procedures, etc.), benefit and confidentiality of the study, after which their written informed consent to participate in the study was obtained.

### 2.2. Sociodemographic and clinical information

Basic sociodemographic information was collected for all study participants, including age, gender, education, marital status (married or unmarried) and occupational status (employed or unemployed); clinical features including first episode or not, age of onset of illness, number of lifetime hospitalizations, and psychotic medication usage were also collected. Additionally, gender, education, first episode or not, age of onset of illness and whether living with others were examined only at baseline, while the rest of the above variables were examined both at baseline and follow-up.

### 2.3. Violent behavior assessment

All the participants and their caregivers were asked whether the participants had engaged in any violent behavior toward others in the past 12 months. Violent behavior was evaluated by the psychiatrists, using a six-level risk assessment scale according to the National Standards for Basic Public Health Services (the Third Edition), which was issued by the Chinese National Health and Family Planning Commission (http://www.nhc.gov.cn/jws/s3578/201703/d20c37e23e1f4c7db7b8e25f34473e1b.shtml). According to the above regulations, the Chinese psychiatrists must apply this six-level risk assessment scale for the management and treatment in patients with severe mental disorders. Level 0: does not conform to any of the following level 1–5 behaviors; Level 1: verbal threats, shouting, but no beating people or smashing objects; Level 2: beating or smashing behavior against property, restricted to the home, can be persuaded to stop; Level 3: serious beating or smashing behavior against property, regardless of occasion, cannot be persuaded to stop; Level 4: continued beating or smashing behavior against property or people, regardless of occasion, cannot be persuaded to stop, including self-injury or suicidal behavior; Level 5: any act of violence against a person with a controlled dangerous weapon, or acts of arson or explosion, whether at home or in a public place. In this study, Level 0 was classified as no violent behavior, while levels 1–5 were classified as presence of violent behavior.

### 2.4. Psychiatric symptom assessment and psychological assessment

The Chinese version of the Brief Psychiatric Rating Scale (BPRS) was used to evaluate the severity of clinical symptoms. The results of total and sub-evaluation of BPRS were very consistent (*r* = 0.85–0.99, *P* < 0.01), and there was a high degree of consistency between the checks-recheck total scores within 3 days (*r* = 0.52, *P* < 0.01), which indicated good reliability. Additionally, there was a high positive correlation between the total score of BPRS and the clinician's judgment of the severity of the disease (*r* = 0.84, *P* < 0.01), and the efficacy assessed by BPRS was basically consistent with the clinical efficacy (*r* = 0.60, *P* < 0.01), which indicated good validity (16, 17). BPRS is a 7-point Likert-type clinician-administered scale, rated from no symptoms (1) to extremely severe (7), and higher scores indicate more severe symptoms.

The Chinese version of the Montgomery–Asberg Depression Rating Scale (MADRS) was used to measure depressive symptoms. The inter-rater reliability of Chinese version of MADRS was 0.954, the Cronbach α coefficient was 0.847, and the criterion related validity with HAMD total score and CGI-S were 0.853 and 0.672, respectively (both *P* < 0.01), indicating good reliability and validity (18, 19). MADRS is a 6-point Likert-type clinician-administered scale. The total score ranges from 0 to 60 points, and higher scores represent more severe depressive symptoms. Meanwhile, we employed the Chinese version of the 16-Item Quick Inventory of Depressive Symptomatology, self-report (QIDS-SR16) to enable the participants to rate their own depressive symptoms. The internal consistency (Cronbach's alpha) ranged from 0.73 to 0.82 for QIDS-SR at both the baseline and exit (6 weeks later). The QIDS-SR total scores were highly correlated with the HAMD total score at both baseline (*r* = 0.54, *p* < 0.01) and exit *r* = 0.72, *p* < 0.01, respectively) (20, 21). This scale consists of 16 items, each of them ranges from 0 to 3 points, and a higher total score indicates greater severity of depressive symptoms.

Insight was measured by the Chinese version of the Insight and Treatment Attitude Questionnaire (ITAQ). The retest reliability was 0.869 (*p* < 0.001), the inter-rater reliability was 0.80, the half-score reliability was 0.903, and the validity between clinical experience of the general evaluation results of insight and BPRS was 0.7075 and −0.40, respectively, indicating that ITAQ has good reliability and validity (22, 23). In the scale, five items assessed patients' awareness of illness, while 6 items assessed patients' awareness of the need for treatment. ITAQ is a clinician-administered scale, in which each item ranges from 0 to 2 points, and a higher total score means better insight.

Sleep status was also evaluated as sleep disturbance may also be relevant to violent behavior (24). We used an eight-question self-report scale to assess the sleep status of the participants over the past month, which is widely used in other Chinese studies (25, 26). The scale included four sub-domains: any type of insomnia [e.g., difficulty initiating sleep (Over the past month, have you had trouble falling asleep), difficulty maintaining sleep (Over the past month, have you had difficulty maintaining sleep for a long time or waking up from time to time?) and early morning wakening [For the past month, have you had trouble waking up in the middle of the night or waking up too early to go to sleep again]; whether insomnia requires treatment [Have you been using sleeping pills (medicines) for insomnia for the past 1 month?]; whether bothered by insomnia [Have you been bothered by insomnia for the past 1 month?]; whether insomnia affects life, work and study [Have you suffered from insomnia that affects life, work, and study for the past 1 month?

The Sheehan Disability Scale (SDS) with good reliability and validity (27) was used to evaluate functional impairment. The internal consistency reliability of the SDS is high, with coefficient alpha of 0.89 and the construct validity was substantiated in two ways (28). SDS is a self-report scale, including three sub-domains of functioning: work/school, social life and family life. The total score ranges from 0 to 30 points, and higher scores represent poorer functioning.

We used the Chinese brief version of the World Health Organization Quality of Life instrument (WHOQOL-BREF) to evaluate health-related quality of life. The scale has good internal consistency, discriminative validity and structural validity. There is a high correlation between the scores in each field of the WHOQOL-BREF and the scores in the corresponding fields of the WHOQOL-100 scale, in which the Pearson correlation coefficient is 0.89 (Social relationships domain) at the lowest and 0.95 (physical health domain) at the highest (29, 30). WHOQOL-BREF consists of 28 items divided into four sub-domains: physical health, psychological health, social relationships and environment. WHOQOL-BREF is a self-report scale, in which each item ranges from 1 to 5 points, and a higher total score means better quality of life.

Treatment satisfaction of the patients, their relatives and doctors were separately measured with a 7-point Likert-type self-report scale, in which the total score ranges from 1 to 7 points, representing the degree of satisfaction from extremely unsatisfactory to extremely satisfactory.

Reliability training was conducted by the three researchers prior to this study, in which 20 patients with schizophrenia were co-rated. The interrater reliability of the rating instruments yielded highly satisfactory agreement (intraclass correlation coefficients and kappa values >0.90).

### 2.5. Statistical analysis

Data were analyzed by IBM SPSS v26.0. The comparisons between the completed follow-up sample and the lost-to-follow-up sample on sociodemographic and clinical characteristics were performed by Pearson chi-square test and Independent-Samples *T*-Test as appropriate. McNemar Test and Paired-Samples *T*-Test were applied to analyze the differences concerning sociodemographic and clinical characteristics between the baseline group and the follow-up group in the 491 participants who completed the follow-up visit.

Subsequently, Generalized Estimating Equations (GEE) (31) were used to analyze the risk factors for violent behavior. GEE is mainly used for the analysis of repeated measurement data, in which there are more flexible observation time points. We conducted only one follow-up visit so far, yet there will be more follow-up visits in the future. Thus, GEE is more suitable for the statistical analysis in the present study. Risk factors were added as independent variables into the GEE analysis based on previous relevant studies, our clinical experience and the results of the bivariate analyses (see Table 2). More precisely, “Baseline variables” mean the variables which would not change over time, which include “Male, First-episode (or not), Education level at baseline” and so on. Besides, “Time-dependent variables” mean the variables which would change over time, including “Married, Employed, On FGAs” and so on. Additionally, in GEE analysis, variables with large correlations would not be selected as independent variables simultaneously. For example, “BPRS Positive, BPRS Negative and BPRS Affect” were chosen as independent variables, while “BPRS Total” not, as the latter was the sum of the former. The level of significance was established at 0.05 (two-tailed).

Effect size was calculated if the result was statistically significant, so as to consider whether the change was also clinically meaningful. Cohen's d value was calculated as the effect size when comparing two mean values, in which 0.2 represents a small effect size, 0.5 represents a medium effect size, and 0.8 represents a large effect size. Phi coefficient was calculated as the effect size when comparing two rates, in which 0.1 represents a small effect size, 0.3 represents a medium effect size, and 0.5 represents a large effect size.

## 3. Results

### 3.1. Sample characteristics and descriptive analyses

There were 491 participants with schizophrenia who took part at both baseline and follow-up 2 years later, giving a response rate of 66.2% at follow-up. Those who dropped out were significantly more likely to be living with others (Phi = 0.07), and significantly less likely to be a current smoker (Phi = 0.08), taking clozapine (Phi = 0.11) and had a smaller number of hospitalizations across their lifetime (Cohen's d = 0.16): however, all four effect sizes were small or very small (see Table 1).

**Table 1:** Comparison of sociodemographic and clinical characteristics between the completed follow-up sample (*n* = 491) and the lost-to-follow-up sample (*n*=251).

	**Completed follow-up sample (*****n*** = **491)**		**Lost-to-follow-up sample (*****n*** = **251)**		**Statistics**		
	***n***	**%**	***n***	**%**	χ^2*$*^	**df**	***p***
Male	313	63.7	149	59.4	1.35	1	0.244
Married	213	43.4	115	45.8	0.40	1	0.527
Employed	318	64.8	170	67.7	0.64	1	0.421
Living with others	453	92.3	241	96.0	3.87	1	**0.049**
First episode	47	9.6	33	13.1	2.207	1	0.137
Current drinker	20	4.1	8	3.2	0.35	1	0.549
Current smoker	122	24.8	44	17.5	5.12	1	**0.024**
Physical disease	27	5.5	22	8.8	2.87	1	0.090
Family psychiatric history	111	22.6	57	22.7	0.001	1	0.975
Violent behavior in past 1 year	205	41.8	109	43.4	0.19	1	0.662
On FGAs	143	29.1	57	22.7	3.47	1	0.062
On clozapine	104	21.2	31	12.4	8.702	1	**0.003**
On SGAs other than clozapine	226	46.0	120	47.8	0.21	1	0.646
On antidepressant	8	1.6	4	1.6	0.001	1	0.971
On benzodiazepine	22	4.5	11	4.4	0.004	1	0.951
On mood stabilizer	58	11.8	23	9.2	1.19	1	0.274
On anticholinergic	164	33.4	81	32.3	0.09	1	0.757
Insomnia in past 1 month	241	49.1	121	48.2	0.051	1	0.821
	**Mean**	**SD**	**Mean**	**SD**	**T** ^%^	**df**	***p***
Age (years)	40.35	11.907	38.86	13.42	−1.53	740	0.124
Education level (years)	8.27	2.16	8.17	2.303	−0.55	740	0.578
Age of onset (years)	26.20	9.18	25.79	9.24	−0.57	740	0.563
Number of lifetime hospitalizations	2.16	1.92	1.80	1.97	−2.34	740	**0.020**
BPRS total	25.89	8.25	26.34	8.29	0.702	740	0.483
BPRS positive	6.12	2.73	6.08	2.55	−0.19	740	0.845
BPRS negative	5.54	2.76	5.50	2.906	−0.201	740	0.841
BPRS affect	5.13	1.72	5.08	1.57	−0.42	740	0.675
MADRS total	4.69	5.70	5.29	5.96	1.33	740	0.182
QIDS-SR16 total	3.55	3.52	3.72	4.09	0.57	740	0.563
ITAQ total	9.83	7.78	9.68	7.72	−0.24	740	0.809
ITAQ illness	3.97	3.38	3.84	3.43	−0.47	740	0.636
ITAQ medication	5.86	4.604	5.84	4.53	−0.05	740	0.953
SDS work/school	4.03	2.19	4.19	2.35	0.93	740	0.351
SDS social life	4.07	2.203	4.25	2.34	1.03	740	0.303
SDS family life	4.02	2.25	4.29	2.402	1.51	740	0.131
Patient satisfaction	4.25	0.97	4.33	1.06	0.92	740	0.356
Relative satisfaction	4.26	1.01	4.30	1.107	0.51	740	0.604
Doctor satisfaction	4.31	1.05	4.31	1.13	0.11	740	0.912
WHOQOL-BREF physical health	12.26	1.41	12.10	1.53	−1.48	740	0.138
	***n***	**%**	***n***	**%**	χ^2*$*^	**df**	***p***
WHOQOL-BREF psychological health	12.53	1.21	12.44	1.402	−0.87	740	0.380
WHOQOL-BREF social relationships	12.10	2.09	11.81	1.87	−1.82	740	0.068
WHOQOL-BREF environment	11.87	1.29	11.84	1.37	−0.22	740	0.823

Bold values: *P* < 0.05; ^*$*^ Pearson chi-square test; ^%^Independent-Samples *T*-Test.

BPRS, Brief Psychiatric Rating Scale; FGAs, First-generation antipsychotics; ITAQ, Insight and Treatment Attitude Questionnaire; MADRS, Montgomery–Asberg Depression Rating Scale; QIDS-SR16, The 16-Item Quick Inventory of Depressive Symptomatology, Self-Report; SGAs, second-generation antipsychotics; SDS, Sheehan Disability Scale; WHOQOL-BREF, The World Health Organization Quality of Life- brief version.

### 3.2. Changes in sociodemographic and clinical variables between baseline and 2-year follow-up

At follow-up, participants were significantly less likely to be employed (Phi = 0.38) and experienced more hospitalization episodes over their lifetime (Cohen's d = 0.73) than at baseline. Compared with at baseline, participants were more likely to report taking clozapine (Phi = 0.25), second-generation antipsychotics (SGAs) other than clozapine (Phi = 0.44), antidepressants (Phi = 0.09), benzodiazepines (Phi = 0.26), mood stabilizers (Phi = 0.21) and anticholinergics (Phi = 0.13) after 2 years. Concerning medication dosage, participants reported taking significantly larger doses of clozapine (Cohen's d = 0.18) and SGAs other than clozapine (Cohen's d = 0.42) after 2 years in comparison with baseline (see Table 2).

**Table 2:** Comparison of sociodemographic and clinical characteristics between baseline and 2-year follow-up in 491 patients with schizophrenia.

	**Baseline**		**Follow-up**		**Statistics**		
	**n**	**%**	**n**	**%**	*χ2^$^*	**df**	***p***
Married	213	43.4	220	44.8	0.43	1	0.510
Employed	318	64.8	190	38.7	70.12	1	**< 0.001**
Violent behavior in past 1 year	205	41.8	198	40.3	0.17	1	0.677
On FGAs	143	29.1	162	33.0	3.02	1	0.082
On Clozapine	104	21.2	164	33.4	29.50	1	**< 0.001**
On SGAs other than clozapine	153	31.2	288	58.7	94.01	1	**< 0.001**
On Antidepressant	8	1.6	19	3.9	4.00	1	**0.043**
On benzodiazepine	22	4.5	75	15.3	34.22	1	**< 0.001**
On mood stabilizer	58	11.8	109	22.2	22.12	1	**< 0.001**
On anticholinergic	164	33.4	203	41.3	7.89	1	**0.005**
Insomnia in past 1 month	241	49.1	232	47.3	0.28	1	0.595
	**Mean**	**SD**	**Mean**	**SD**	*T* ^%^	**df**	***p***
Number of lifetime hospitalizations	2.16	1.92	3.06	2.57	−16.23	490	**< 0.001**
PDD/DDD FGAs	0.15	0.35	0.17	0.44	−0.35	490	0.720
PDD/DDD Clozapine	0.09	0.22	0.14	0.26	−4.03	490	**< 0.001**
PDD/DDD SGAs other than clozapine	0.31	0.56	0.65	0.74	−9.25	490	**< 0.001**
BPRS total	25.89	8.25	24.96	6.38	2.15	490	**0.032**
BPRS positive	6.12	2.73	5.85	2.38	1.86	490	**0.062**
BPRS negative	5.54	2.76	5.00	2.23	4.002	490	**< 0.001**
BPRS affect	5.13	1.72	5.54	1.68	−3.94	490	**< 0.001**
MADRS total	4.69	5.70	5.77	6.01	−3.04	490	**0.002**
QIDS-SR16 total	3.55	3.52	5.82	3.69	−10.47	490	**< 0.001**
ITAQ total	9.83	7.78	9.47	6.38	0.95	490	0.342
ITAQ illness	3.97	3.38	3.43	2.81	3.29	490	**0.001**
ITAQ medication	5.86	4.604	6.04	3.92	−0.81	490	0.416
SDS work/School	4.03	2.19	4.10	3.73	−0.35	490	0.720
SDS social life	4.07	2.203	4.07	3.66	−0.02	490	0.983
SDS family life	4.02	2.25	2.97	2.901	7.16	490	**< 0.001**
Patient satisfaction	4.26	0.97	4.70	1.19	−6.67	485	**< 0.001**
Relative satisfaction	4.26	1.02	4.86	1.20	−8.45	466	**< 0.001**
Doctor satisfaction	4.31	1.05	4.4	1.13	−1.66	490	0.098
WHOQOL-BREF physical health	12.26	1.41	13.92	1.73	−17.604	490	**< 0.001**
WHOQOL-BREF psychological health	12.53	1.21	12.57	1.98	−0.42	490	0.668
WHOQOL-BREF social relationships	12.10	2.09	12.77	2.38	−4.87	490	**< 0.001**
WHOQOL-BREF environment	11.87	1.29	12.41	1.76	−5.87	490	**< 0.001**

Bold values: *P* < 0.05; ^*$*^ McNemar Test; ^%^ Paired-Samples *T*-Test.

BPRS, Brief Psychiatric Rating Scale; FGAs, First-generation antipsychotics; ITAQ, Insight and Treatment Attitude Questionnaire; MADRS, Montgomery–Asberg Depression Rating Scale; PDD/DDD, Prescribed Daily Dose/Defined Daily Dose; QIDS-SR16, The 16-Item Quick Inventory of Depressive Symptomatology, Self-Report; SGAs, Second-generation antipsychotics; SDS, Sheehan Disability Scale; WHOQOL-BREF, The World Health Organization Quality of Life- brief version.

With regard to their mental health, participants scored significantly lower at follow-up in the BPRS Total (Cohen's d = 0.10), BPRS Positive (Cohen's d = 0.08) and BPRS Negative (Cohen's d = 0.18), indicating less general and positive as well as negative symptoms; whilst scoring higher in the BPRS Affect (Cohen's d = 0.18), MADRS Total (Cohen's d = 0.14) and QIDS-SR16 Total (Cohen's d = 0.47) than at baseline, indicating more affective symptoms, especially depressive symptoms. Furthermore, follow-up scores were lower in the ITAQ Illness subscale (Cohen's = 0.15) compared to baseline, indicating poorer insight into illness (see Table 2).

However, better patient satisfaction (Cohen's d = 0.30) and better relative satisfaction (Cohen's d = 0.39) regarding treatment were observed at follow-up. Scores at follow-up were lower in the SDS Family life domain (Cohen's d = 0.32) compared to baseline, indicating better family functioning. In addition, follow-up scores in the WHOQOL-BREF Physical health (Cohen's d = 0.79), WHOQOL-BREF Social relationships (Cohen's d = 0.22) and WHOQOL-BREF Environment (Cohen's d = 0.27) domains were higher in comparison with baseline, indicating better health-related quality of life (see Table 2).

### 3.3. Risk factor for violent behavior (GEE analysis)

GEE analysis suggested that for those on clozapine, the likelihood of violent behavior is decreased by 31% (*OR* = 0.69). Each additional point on ITAQ Medication also decreased the likelihood of violent behavior by 9.3% (*OR* = 0.907). In short, use of clozapine and better insight into the need for medication were the protective factors for violent behavior in participants with schizophrenia (see Table 3).

**Table 3:** Generalized Estimating Equations (GEE) results modeling risk factors associated with violent behavior in 491 patients with schizophrenia across 1 year.

**Baseline variables*^*$*^***	**Coef**	**SE**	***P***	**OR**	**95*%CI (OR)***
Male	0.17	0.17	0.323	1.19	0.84–1.68
Living with other at baselines	0.11	0.27	0.661	1.12	0.66–1.92
First episode	−0.14	0.26	0.597	0.86	0.51–1.46
Education level at baseline (years)	0.06	0.03	0.070	1.06	0.99–1.14
Age of onset (years)	−0.01	0.009	**0.049**	0.98	0.96–1.00
**Time-dependent variables** *^%^*					
Married	−0.15	0.17	0.369	0.85	0.61–1.202
Employed	0.11	0.14	0.424	1.12	0.84–1.504
On FGAs	−0.29	0.18	0.118	0.74	0.51–1.07
On Clozapine	−0.36	0.17	**0.034**	0.69	0.49–0.97
On SGAs other than clozapine	0.37	0.15	**0.019**	1.45	1.06–1.98
On Antidepressant	1.14	0.42	**0.007**	3.12	1.35–7.208
On Benzodiazepine	−0.31	0.22	0.156	0.72	0.47–1.12
On Mood Stabilizer	0.58	0.18	**0.001**	1.802	1.26–2.56
On Anticholinergic	−0.28	0.17	0.090	0.74	0.53–1.04
Insomnia in past 1 month	0.32	0.14	**0.025**	1.38	1.04–1.84
Number of lifetime hospitalizations	0.05	0.03	0.103	1.05	0.98–1.13
BPRS positive	0.11	0.03	**0.001**	1.12	1.04–1.19
BPRS negative	−0.03	0.03	0.327	0.96	0.904–1.03
BPRS affect	0.09	0.05	0.075	1.104	0.99–1.23
MADRS total	0.01	0.01	0.270	1.01	0.98–1.05
ITAQ illness	0.01	0.04	0.794	1.01	0.92–1.103
ITAQ medication	−0.09	0.03	**0.004**	0.907	0.84–0.96

Bold values: *P* < 0.05; ^*$*^the value of baseline variables would not change during the whole follow-up period; ^%^the value of time-dependent variables would change during follow-up period.

BPRS, Brief Psychiatric Rating Scale; FGAs, First-generation antipsychotics; ITAQ, Insight and Treatment Attitude Questionnaire; MADRS, Montgomery–Asberg Depression Rating Scale; SGAs, Second-generation antipsychotics.

In contrast, each additional point on BPRS Positive increases the likelihood of violent behavior by 12% (*OR* = 1.12), and for those with insomnia, the likelihood of violent behavior increases by 38% (*OR* = 1.38). Those on SGAs other than clozapine experienced a 45% increase in the odds of violent behavior (*OR* = 1.45), and those on antidepressants were more than three times as likely to report engaging in violent behavior (*OR* = 3.12), while those on mood stabilizers had an 80% increase in the odds of violent behavior (*OR* = 1.802). In short, higher positive symptoms, insomnia, as well as use of SGAs other than clozapine, use of antidepressants and use of mood stabilizers were the risk factors for violent behavior in these participants (see Table 3).

## 4. Discussion

This study found that about two in five community-dwelling patients with schizophrenia reported violent behavior in the past year. Bivariate analysis showed that at follow-up, participants were significantly less employed, experienced more lifetime hospitalization episodes, reported taking more psychotropic medication, and experienced more depressive symptoms, while having better health-related quality of life (especially in physical health domain) than at baseline. GEE analysis showed that use of clozapine and better insight into medication decreased the possibility of violent behavior, while more severe positive symptoms, insomnia, as well as use of second- generation antipsychotics other than clozapine, antidepressants and mood stabilizers increased the probability of violent behavior.

The drop-out rate of the present study is approximately 33.8%, which is larger than the common acceptable value (< 20%) (32). We have tried many efforts to increase the response rate during follow-up period, still the drop-out rate was relatively high. Therefore, we made comparison on variables between the lost-to-follow-up sample and the completed follow-up sample, to find out whether there was large difference between these two samples. Fortunately, those who dropped out were significantly different from participants who completed follow-up in only 4/43 examined variables, and meanwhile the four effect sizes were all small or very small, which suggested those differences may not be clinically meaningful and that the completed follow-up sample may be broadly representative of the whole sample.

We have reported relatively high rates of violent behavior over the past year for the follow-up participants, whether at baseline (41.8%) or 2 years later (40.3%). Reported rates of violent behavior have differed across studies: for instance, a review involving 31 studies showed that outpatients with severe mental illness reported rates of 2%–13% of violent perpetration in the past 6 months to 3 years (33), while other studies reported rates of 14–20% (34–36). The above inconsistent findings may possibly be explained by differences in sample size, the research subjects of interest, the definition and the time period of violent behavior. After a 2-year follow-up period, more participants with schizophrenia were admitted to the hospital and reported taking more, and higher doses of psychotropic drugs, especially SGAs other than clozapine, than at baseline. This suggests worsening of their illness over time, requiring more medical services and medication. Additionally, at follow-up, participants had a higher and probably meaningful rate of unemployment than at baseline, which may be explained by their continuing hospitalizations or accumulated experiences of stigma and/or depression.

Depression is commonly seen in all stages of schizophrenia (37), and is associated with serious consequences in patients with schizophrenia (38). Interestingly, in our study, the scores of BPRS Affect, MADRS Total and QIDS-SR16 Total increased compared with baseline, although only the effect size of QIDS-SR16 Total was close to medium effect size. This might indicate that clinicians should pay more attention to the aggravation of depressive symptoms in patients with schizophrenia, especially considering that the QIDS-SR16 was the only self-report measure. Moreover, health-related quality of life (especially in the subdomains of physical health, social relationships and environment) increased in these participants with schizophrenia after 2 years, although only the effect size of WHOQOL-BREF Physical health is close to a large effect size. Our observed decrease in positive and negative symptoms, especially likely to be associated with increased usage of clozapine, may partly account for this. Similarly, Verma's study found that treatment with clozapine leads to improvement in core symptoms of schizophrenia and is also associated with significant improvement in the quality of life (39).

We applied GEE analysis to identify the risk factors for violent behavior in participants with schizophrenia. Our study indicated that more severe positive symptoms in participants with schizophrenia related to more violent behavior, which was similar to the finding of another study (40), whose results showed that the most predictive variables for violence among inpatients with schizophrenia are suspiciousness and hostility, more severe hallucinations, poor insight into delusions and the overall illness, and greater disorganization of thought processes (40).

Otherwise, any type of insomnia (including difficulty initiating sleep, difficulty maintaining sleep and early morning wakening) in the past month was also a risk factor, in line with a systematic review, which reported a relationship between interpersonal violence and a broad range of sleep disturbance, whereby 46 to 100% of respondents endorsed moderate to severe insomnia (24). Symptoms of sleep disruption can predict the onset of positive psychotic symptoms, such as paranoia and hallucinations. With sleep disturbances inextricably linked to increased severity of schizophrenia and worsening clinical outcomes, insomnia is an important therapeutic target within this patient population (5, 41).

Unlike many other studies, this study explored in depth the relationship between psychotropic drugs and violent behavior in participants with schizophrenia. We found that clozapine usage was a protective factor, while usage of SGAs other than clozapine was a risk factor. Whilst all SGAs are prescribed for severe and/or ongoing psychotic symptoms, it is recognized that clozapine has greater efficacy than other SGAs due to its high affinity for dopamine 4 vs. dopamine 2 receptors (42), which might explain our findings. Furthermore, clozapine has also shown the strongest evidence for treating acute violence in schizophrenia (43). Thus, our finding may indicate that clozapine should be preferred to reduce acute aggression and control persistent violence in patients with schizophrenia rather than other SGAs and FGAs (44), especially in refractory conditions (45).

Use of antidepressants by participants showed a 3-fold association with violent behavior. The previously described worsening of depressive symptoms in participants with schizophrenia after 2 years follow-up may explain the increase in antidepressant usage. It is well-known that irritability associated with depression and anxiety could culminate in aggression (46). There is reason to believe that the depression reported here is under-treated and hence irritability leads to aggression, thus increasing the risk for violent behavior in participants with schizophrenia.

Besides, we found that use of mood stabilizers was also a risk factor, as increased mood stabilizer usage itself may manifest because of a more serious disease condition. Compared with our study, previous studies discovered that lithium has been repeatedly shown to reduce irritability and incidents of aggression in bipolar disorder patients (45), while valproate promoted significant reductions in aggression across multiple diagnostic categories (47). It could also be possible that the mood disturbance was under-diagnosed and under-treated in this sample, therefore leading to inconsistent results compared to other studies.

The strengths of this study were as follows: (a) compared to the many cross-sectional studies published before, a follow-up study is a more robust design for this enquiry, in which we could explore the complete development process and some key turning points in the development process. (b) GEE analysis (rather than traditional logistic regression analysis) was applied to identify the risk factors for violent behavior, by which we could effectively explore the changes of sociodemographic and clinical characteristics during the follow-up period that affect the outcome (rather than just explore baseline or endpoint variables). (c) participants in this study were patients with schizophrenia managed in primary mental health care in an under-developed city in China, from which the research findings could be applied to other similar developing regions.

The limitations of this study were as followed: (a) attrition bias: the drop-out rate of this follow-up study is relatively high (about 33.8%), and we only included participants from one rural city (Luoding city), which may have impacted the generalizability of the findings. (b) Follow-up study is a kind of descriptive study, which can only propose etiological hypotheses and clues, rather than determine causal relationship. (c) we only assessed whether the study participants were engaged in violent behavior in the past year or not, rather than recorded different degrees of severity and frequency of violent behaviors, which prevents fine-grained analysis of differences in frequency and severity. In the future follow-up, we will perform stratified analysis to deeply explore the status and characteristics of violent behaviors. (d) stigma, discrimination, and response bias (such as social desirability) may have influenced the accuracy of the results, as some of the scales are self-report. Further, participants reporting to the interviewers with or without the presence of relatives may also influence the results. (e) only individual factors were explored in the present study, whilst social or environmental or system level factors were not included, which are also critical to the occurrence of violent behavior. (f) we only conducted one follow-up till now, which limited the findings of the study, yet there will be on-going follow-ups in the future.

To conclude, risk evaluation, prevention and management of violence are demanded as part of the completed patient assessment and treatment in primary psychiatric practice or other settings. Psychiatrists should pay more attention to the irritability associated with depression and mood disturbance and give priority to the usage of clozapine, as appropriate, in patients with schizophrenia with severe or treatment-refractory illness. They should also evaluate and treat any insomnia experienced by their patients. Otherwise, both primary care practitioners and families should also concern more on the above insomnia problem, and seek help from psychiatrists if necessary.

## Data availability statement

The original contributions presented in the study are included in the article/supplementary material, further inquiries can be directed to the corresponding author.

## Ethics statement

The studies involving human participants were reviewed and approved by the Clinical Research Ethics Committee of Guangdong Provincial People's Hospital. The patients/participants provided their written informed consent to participate in this study.

## Author contributions

Study design: C-LH, S-BW, and CH. Data collection and typing: Z-HH, FW, Q-WW, X-YH, Z-LC, and Y-NX. Analysis and interpretation of data and drafting of the manuscript: Z-HH, FW, and C-LH. Critical revision of the manuscript: C-LH, CH, CM, S-BW, Z-LC, and Y-NX. Approval of the final version for publication: All coauthors. All authors contributed to the article and approved the submitted version.
